# Projections and the Potential Societal Impact of the Future of Neurotechnologies

**DOI:** 10.3389/fnins.2021.658930

**Published:** 2021-11-15

**Authors:** Kate S. Gaudry, Hasan Ayaz, Avery Bedows, Pablo Celnik, David Eagleman, Pulkit Grover, Judy Illes, Rajesh P. N. Rao, Jacob T. Robinson, Krishnan Thyagarajan, The Working Group on Brain-Interfacing Devices in 2040, Nena Bains

**Affiliations:** Kilpatrick Townsend & Stockton LLP; Kilpatrick Townsend & Stockton LLP; Avalere Health; Rice University; Kilpatrick Townsend & Stockton LLP; Kilpatrick Townsend & Stockton LLP; Emory University Center for Ethics, Neuroethics Progam; Kilpatrick Townsend & Stockton LLP; ^1^Kilpatrick Townsend & Stockton LLP, Washington, DC, United States; ^2^School of Biomedical Engineering, Science and Health Systems, Drexel University, Philadelphia, PA, United States; ^3^Department of Psychology, College of Arts and Sciences, Drexel University, Philadelphia, PA, United States; ^4^Drexel Solutions Institute, Drexel University, Philadelphia, PA, United States; ^5^Department of Family and Community Health, University of Pennsylvania, Philadelphia, PA, United States; ^6^Center for Injury Research and Prevention, Children’s Hospital of Philadelphia, Philadelphia, PA, United States; ^7^Substrate Group, New York, NY, United States; ^8^Department of Physical Medicine and Rehabilitation, Johns Hopkins, School of Medicine, Baltimore, MD, United States; ^9^Department of Psychiatry, Stanford University School of Medicine, Stanford, CA, United States; ^10^Center for the Neural Basis of Cognition, Carnegie Mellon University, Pittsburgh, PA, United States; ^11^Department of Electrical and Computer Engineering, Carnegie Mellon University, Pittsburgh, PA, United States; ^12^Department of Medicine, University of British Columbia, Vancouver, BC, Canada; ^13^Neuroethics Canada, University of British Columbia, Vancouver, BC, Canada; ^14^Center for Neurotechnology, Paul G. Allen School of Computer Science and Engineering, University of Washington, Seattle, DC, United States; ^15^Department of Bioengineering, Rice University, Houston, TX, United States; ^16^Department of Electrical and Computer Engineering, Rice University, Houston, TX, United States; ^17^Applied Physics Program, Rice University, Houston, TX, United States; ^18^Department of Neuroscience, Baylor College of Medicine, Houston, TX, United States; ^19^Palo Alto Research Center (PARC), A Xerox Company, Palo Alto, CA, United States

**Keywords:** ethics, neuroethics, brain interfacing, policy, brain recording, brain stimulation, non-invasive, minutely invasive

## Abstract

Traditionally, recording from and stimulating the brain with high spatial and temporal resolution required invasive means. However, recently, the technical capabilities of less invasive and non-invasive neuro-interfacing technology have been dramatically improving, and laboratories and funders aim to further improve these capabilities. These technologies can facilitate functions such as multi-person communication, mood regulation and memory recall. We consider a potential future where the less invasive technology is in high demand. Will this demand match that the current-day demand for a smartphone? Here, we draw upon existing research to project which particular neuroethics issues may arise in this potential future and what preparatory steps may be taken to address these issues.

## Introduction

Capabilities of today’s most powerful brain-interfacing technologies are extraordinary. Brain stimulation can alter a person’s memory (Beynel et al., 2019; Reinhart and Nguyen, 2019), attentiveness (Filmer et al., 2017; Curtin et al., 2019), mood (Mayberg et al., 2005; Schlaepfer et al., 2008), and physical capabilities (Wagner F. B. et al., 2018; Barbe et al., 2020). Brain recordings can allow sensed stimuli, perceptions and motor intentions to be decoded (Kay et al., 2008; Edelman et al., 2015; Gateau et al., 2018; Liu and Ayaz, 2018; Sani et al., 2018; Volkova et al., 2019; Krol et al., 2020; Schwarz et al., 2020). Yet, to date, the most dramatic stimulation-triggered actions and the most temporally and spatially precise recordings primarily use very invasive technologies (Lebedev and Nicolelis, 2017; Wallis, 2018). Invasive technology currently faces impediments about the potential limitations of adoption, the potential of adverse events (from implantation surgery or adverse events from usage, such as the possibility of burns), the potential reduced quality of recorded neurological signals over time, and the potential reduction in impact of stimulation over time. Development of non-invasive neurotechnologies is progressing rapidly and demonstrating potential beyond research toward everyday life (Roelfsema et al., 2018; Dehais et al., 2020). And, even today, some commercial home-use consumer devices are already on the market (Ienca et al., 2018).

Might the capabilities of brain-interfacing technology advance sufficiently to garner demand akin to the modern-day smart phone? If so, what policy issues might this technology present to society, and how might we prepare for this potential future.

### Present-Day Brain-Interfacing Technology

Advances in neuroscience and engineering have facilitated development of diverse non-invasive neurotechnologies for monitoring and modulating brain activity (Roelfsema et al., 2018). Whole-brain activity monitoring modalities [e.g., functional magnetic resonance imaging (fMRI) or magnetoencephalogram] require room-size equipment but provide high spatial resolution (though portable MRI is being increasingly explored). Portable recording modalities [e.g., electroencephalography (EEG) and near-infrared spectroscopy (NIRS)] have lower spatial resolution but are widely used to study neural mechanisms underlying cognitive functioning within real-world contexts (Ayaz and Dehais, 2019). Non-invasive brain-stimulation (NIBS) [e.g., transcranial magnetic and electrical stimulation (TMS, tES)] is used for research, prognostication and treatment of many disorders (Bikson et al., 2020). Focused ultrasound (FUS) is emerging as a high-resolution and potentially portable alternative, pending safety challenges (Shen et al., 2020). Targeted indirect brain modulation even may be achieved *via* visual sensory substitution (Adaikkan and Tsai, 2020) and somatosensory senses (Novich and Eagleman, 2015).

Non-invasive neurotechnology already has been used to restore function or enhance human capabilities, including motor abilities, communication, perception, attention, mood, situational awareness, memory, problem-solving, and decision making (Cinel et al., 2019). TMS is FDA-approved to treat major depression and obsessive-compulsive disorder. Other non-invasive neurotechnologies have tracked speaker-listener communication (Liu et al., 2017) and decoded participants’ mental states (Trimper et al., 2014), and supported brain-to-brain communication between multiple brains (Jiang et al., 2019).

Nonetheless, to date, invasive neurotechnology maintains the highest spatial and temporal resolution, deep-brain accessibility and performance. Established forms of such technologies include electrocorticography (ECoG); multi-unit electrode arrays and tetrodes; and emerging ultraminiature and flexible technologies, with spatial resolution reaching sub-50 micron (Neely et al., 2018). These technologies have prompted invasive-device use for augmentative applications, such as communication *via* translating cortical activity to text, mood regulation, quicker memory recall and brain co-processors (Ezzyat et al., 2018; Hampson et al., 2018; Rao, 2019; Shanechi, 2019; Makin et al., 2020). Although invasive technologies carry many risks (e.g., brain tissue damage associated with surgery, infection, implantation, and explantation) (Hendriks et al., 2019), they currently provide the fastest operation and greatest portability, in addition to the highest spatiotemporal resolution. As improved non-invasive technologies become more competitive, development focus has shifted to improving perceived benefits relative to accompanying risks.

### State of the Art and Engineering

Recent engineering breakthroughs suggest that, non-invasive or minutely invasive portable wireless technologies will soon record from 50,000 to 100,000 neurons simultaneously (with minutely invasive devices being temporarily and non-surgically provided to the brain). This projection is based on prior exponential scaling (Stevenson and Kording, 2011) of the number of neurons simultaneously recorded. These interfaces likely will be able to detect dendritic/axonal level activity and record and affect neurotransmitters and ion concentrations that drive neural behavior. The feasibility of achieving these capabilities is evidenced by a recent DARPA program called the Next-Generation Non-surgical Neurotechnology (N^3^) program, which seeks to develop non-invasive or minutely invasive interfaces having 50-ms temporal resolution and 1-mm^3^ spatial resolution for closed-loop sensing and stimulation from each of 16 or more brain locations.

**FIGURE 1:**
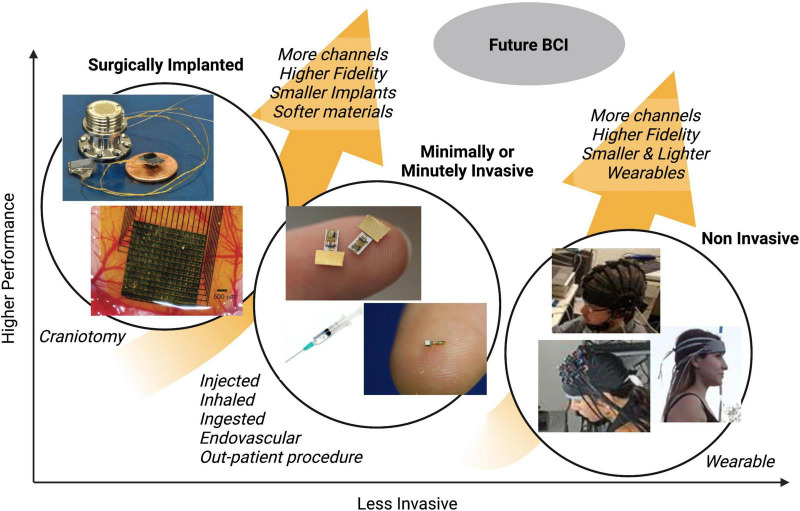
Illustration of how different types of neurotechnology generally vary with respect to performance and invasiveness.

Figure 1 illustrates how different types of existing neurotechnologies vary with respect to invasiveness and performance metrics (e.g., spatial and temporal precision). As shown, minimally invasive devices (which can be used without a user having a craniotomy), and minutely invasive devices (which can be used without a user having an incision), are less invasive than surgically implanted devices but more invasive than wearable devices. Technological advances are currently and likely to continue to trend toward improved performance and reduced invasiveness. The future of brain-interfacing devices, therefore, may be sufficiently non-invasive to not require surgery, but still be capable of recording or stimulating brain areas with high temporal and spatial precision.

In our view, the goals of the DARPA N^3^ grant and others similar to it will be achieved or surpassed by 2040. We project that there will be high demand for non- or minutely invasive brain-recording devices that have capabilities that include at least one of: enhancing attention, memory or learning; enhancing mood; or supporting inter-person communication. This projection of high demand is further based on a projection that having the technical capabilities of recording from and/or stimulating brain regions in a temporally and spatially precise manner will facilitate further understandings of human neuroscience, such that such recordings or stimulations provide a practical use. Given these projections, we consider key ethical implications here. Some are commensurate with past discussions about neurotechnology; others are novel.

## Discussion of Neuroethical Issues of the Potential Brain-Interfacing Future

### Access

Minutely invasive or non-invasive brain interfaces that safely enhance brain function could be advantageous in some academic, recreational and professional settings. Well-resourced societies may actually deem technology to be as essential to learning and job performance as computers are now. Schools and employers may routinely supply brain-interfacing devices. However, as with other health or performance enhancing products, such provisions may result in disparate access that exacerbates existing disparities. Further, if global initiatives are not established to provide equal access to brain-interfacing technology, global inequalities and instability are likely to become even more pronounced than they are today (Delegates et al., 2018). Access to technology that can alter brain function in ways that enhance productivity can further, and arguably in more significant ways, exacerbate global inequity and socioeconomic divides.

The achievability of fair access may depend on a degree to which potential users trust that recorded neural data will be secure (e.g., from the government or from being sold or availed to corporations) and stimulation will only be of a type for which a user provided informed consent. Toward this goal, we suggest three anticipatory remedies:

1. Government establishment of distributions, subsidizations, incentive programs, to facilitate access to brain-interfacing devices across populations.
2. Shaping of marketing, price points, regulation and education by technologists and policy-makers to promote, not only fair device access, but also widespread understanding of the potential value and risks of technology. Such efforts can support underprivileged individuals while also improving the productivity and well-being of societies.
3. Passing of regulations that restrict the authority of a government or corporations to receive raw or processed brain data or to control stimulation by designing networks that thwart unauthorized access, supporting watchdog entities, and publicizing these outreach efforts.
4. Evaluation of cultural meaningfulness and receptivity to brain technologies on a case-by-case basis, as traditional and biomedical understandings of brain wellness, enhancement, and disease are far from homogeneous (Delegates et al., 2018).

Despite such efforts, some potential users or populations may remain skeptical of potential unauthorized use or control and may reject the technology. Much of this process will require cultivation of scientists’ and engineers’ orientation toward voices and needs of the end-user (Sullivan et al., 2017). Ultimately, decisions to reject the technology must be respected by society.

### Power Asymmetries in the Workplace or Militaries

Due to the potential performance enhancements, it is conceivable that employers or governments may implement policies requiring, availing or prohibiting use of neurotechnologies. For example, many employers currently require computer-based augmentation of their employees’ capabilities and offer caffeine stimulation. If neurotechnology proves to improve capabilities, this technology may conceivably become an explicit or implicit job requirement (Bard et al., 2018; Brühl et al., 2019; Dubljevic et al., 2019), and ethical considerations of the usage of human-integrated technology may be all the more important (Gauttier, 2019).

Currently, most risks of job-associated technologies are well-characterized (e.g., consider risk of factory workers vs. SWAT teams). However, very few studies have explored whether usage of a brain-interfacing device for hundreds of hours per month (particularly when used for enhancement, and not medical, purposes). For example, such usage may present health risks, confusion of users of body integrity, shifts in users’ identities as individuals, and/or pressure to use neurotechnology intensely to keep with a raised society-, employer-, or individual-imposed performance bar. Therefore, we recommend that:

1. Studies be conducted that identify any biological risks of extended use of brain-interfacing devices (e.g., 50 + *hour* work-weeks for years) (Sahakian et al., 2015), which can inform the decisions of government agencies, soldiers, employers and employees as to what type of usage of brain-interfacing devices is reasonable.
2. Information-distribution campaigns be initiated to ensure that information that details any potential risks or uncertainties is availed to employees or soldiers who may be asked by their employers or superiors to use brain-interfacing devices and to employers so as to understand the potential impacts and uncertainties of requests to use brain-interfacing devices. A focus in the distribution of responsibilities that lie with an individual vs. an institution (e.g., school or military) may further help individuals understand how to plan for (e.g., train for and/or deliberate about) particular potential circumstances that may arise and to assess risk of the technology’s use (Binnendijk et al., 2020).

### Consent/Assent

If non-invasive or minutely invasive brain-interfacing technology advances in its capabilities (in a manner where significant side effects are not observed), parents may believe that brain-interfacing technology will promote their children’s success. For example, conceivably, non-invasive or minutely invasive brain-interfacing technology may be used to help to establish sleep routines, deliver personalized education, and even provide the opportunity to control toddlers’ outbursts, However, brain-recording data may reflect mere contemplations or self-identifications that may correspond to most-personal data, and parents and children may be at odds about what degree of stimulation-based enhancement is desirable (Maslen et al., 2014). Further, long-term impacts of the use of brain-interfacing technology on the pediatric population may be inadvertently overlooked.

Therefore, we suggest:

1. Child advocates be intensively engaged when determining what types of brain-interfacing usage is permitted or required for children.
2. Standards or laws be established that define limits on parental access and control of neural data and stimulation.
3. Long-term pediatric studies also be performed to alert society of whether and how stimulation affects brain plasticity, induces addiction, and alters neural development, and long-term risks. Depending on studies’ outcomes, society may choose to restrict pediatric use (e.g., as prescriptions are now) or may largely rely on sound parental judgment (e.g., consider the accessibility of coffee).

Assessments relating to the pediatric population and the potential of future non- or minutely invasive brain-interfacing technology extends beyond consent and assent issues (e.g., potential for discrimination, potential for evading best interests of a child, etc.) (Dubljevi, 2019; Committee on the Rights of the Child [CRC], 2021). At least some of the suggested approaches above may facilitate informed decision-making on multiple issues relating to the intersection of the pediatric population and the potential future of non- or minutely invasive brain-interfacing technology.

### Data Privacy, Security, and Liability

Currently, laws, professional standards, and regulations. Exist to ensure that medical data is protected and that informed consent it obtained before performing medical procedures. For example, the U.S. Health Insurance Portability and Accountability Act (HIPAA) includes a Privacy Rule that includes restriction of when protected health information can be released to a party. However, many of these laws, standards, and regulations (e.g., HIPAA) are focused on medical or investigational use cases and may not pertain to uses of non-medical uses of brain-interfacing technology. Some other laws, professional standards, and regulations [e.g., the European General Data Protection Regulation (GDPR)] focus on protecting individuals’ personal data, though various criteria still differentially pertain to health data and other data. For example, while “explicit consent” is required for use of health data, only “consent” is required for other data.

Brain-interfacing devices are unique in that this recorded data may well be considered not to be medical or health data (and may thus may not qualify for protections offered by some current laws); scientists may learn to extract more brain-signal information post-recording than originally identified for a specific use; and brain stimulation may alter users’ behavior or personalities (Minielly et al., 2020a; Naufel and Klein, 2020).

#### Recording

Storing neural data can provide value both to the subject and to a larger population by enabling post-collection analysis of the data. However, the longer that the data are stored (and the larger a collective data is) the higher the risk is for unauthorized data access. In view of this risk, we recommend:

1. Signal processing and deletion of the raw brain data be performed expediently. These practices can drastically reduce risks of hacking and unauthorized data use.
2. Data sharing be performed only after informed consent has been received and be limited in content (e.g., restricted to specific brain-region channels, time periods and higher-level variables established by standards). This approach is particularly valuable because expedient data processing and deletion becomes more complicated if a first entity controls neural data initially collected from a device and other entities develop applications to process the brain recordings. Informed consent and imposed limitations of data sharing may result in brain-app developers then sharing in the obligation of expediently processing signals and deleting underlying data.
3. Data-restriction standards and regulations can constructively formally establish which entities own data in which contexts (veering largely to the recorded individual) (Minielly et al., 2020b). Given that device functionalities may well be dependent on knowledge of neural representations of external stimuli and meaningful translations of various types of brain stimuli, we recommend establishing standards that further promote (or require) sharing of raw or processed data. This sharing may avoid the necessity to re-learn user-specific information upon device transfer and may promote efficient data-collection/processing pipelines.

#### Stimulation

In many contexts, legal systems are structured to allow users to choose to take calculated risk. However, these systems are largely premised on the understanding that the users are aware of the potential risks. If non-invasive or minutely invasive brain-interfacing technology will become increasingly common, it is possible that conveying risks to users will require more effort and more explicit warnings. Therefore, we recommend:

1. Guidelines and laws be established to ensure that suppliers of stimulation devices fully inform (Suthana et al., 2018; Wexler and Reiner, 2019) users of stimulation sites, intensity, duration, purposes and onset conditions that are being used for clinical and non-clinical applications.
2. Disclosures clearly convey potential side effects, including long-term use risks (Minielly et al., 2020a).

### Opt-in Default

How can companies obtain informed consent to store neural signatures, and mine, share or sell brain data? Best practices from genetic sequencing companies offer guidance, although brain data presents new challenges. For example, brain data are arguably a closer representation of who a person is than the genome, as it represents not just genetics but also experience (Purcell and Rommelfanger, 2015; Ienca et al., 2018). Additionally, the brain may be quicker to adapt to dynamic changes than even the epigenome. Data from a previous time point might be of questionable relevance to later situations (Eagleman, 2020).

Accordingly, we recommend:

1. Manufacturers and sellers err toward providing and emphasizing potential risks, and government agencies err toward requiring risk disclosure. Risks may involve health risks (e.g., associated with stimulation), identity risks, and the potential of unauthorized data access (e.g., *via* hacking). Given that it may be appropriate to disclose a sizable number of risks, we recommend that risks that are of higher potential magnitude be particularly emphasized.
2. When possible, data controllers (and stimulation controllers) use opt-in instead of opt-out techniques. Requiring opt-in authorization can facilitate ensuring that users understand the potential risks of a given action.

### Regulations, Laws, and Standards

The breadth of possible brain-interfacing devices poses a challenge for government oversight of this technology (Coates McCall et al., 2019). For example, the U.S. Food and Drug Administration (FDA) currently regulates medical devices but not low-risk devices used for entertainment or wellness (e.g., mental acuity or relaxation). The FDA frequently turns to marketing materials for devices to characterize intended use. Currently, many non-invasive brain-interfacing devices used by the general public are not FDA-regulated. Even if they were, they may be exempt from the agency’s premarket notification requirement that assesses safety and efficacy, as demonstrated by the current exemption for EEG devices. Unlike drugs, devices highly similar to pre-approved devices enjoy a low bar of approval.

As the brain interfaces industry grows, we recommend that standards for a variety of neurotechnologies be established to ensure operational performance, conformity, and safety of new systems. New laws will be needed to identify liability: e.g., when is a manufacturer, employer, or user liable for unintended consequences of brain stimulation? Is the user or device manufacturer liable for actions resulting from a brain interface and user co-adapting to each other? If brain signals from a first person’s brain generate stimulation of a second person’s brain, when might the second person be liable for the second person’s actions and when might the first person be liable for the second person’s actions (Maslen et al., 2014).

Historical data from developmental trajectories of many other domains (e.g., ranging wireless communications to equal opportunity in employment) demonstrate that standards and laws catalyze innovation and industry growth. Currently, no existing standards or guidelines exist for brain interfacing products and their system-level function, but a new IEEE Standards Association effort reported a roadmap for brain-machine interfacing standards (The Group on Neurotechnologies for Brain-Machine Interfacing, 2020). However, all stakeholders must participate to converge toward standards that facilitate transparency, interoperability, interpretability, reproducibility, safety, and efficacy.

## Additional Issues and Perspectives

While this work identifies exemplary non- and minutely invasive brain-interfacing technology currently in existence, exemplary research efforts in this field, exemplary neuroethical considerations, and exemplary potential strategies for addressing these considerations, it will be appreciated that the description in each of these areas is incomplete. For example, this publication emphasizes potential neuroethical considerations and strategies that may pertain to a future where the potential neurotechnology identified in the DARPA N^3^ grant (non- or minutely invasive brain-interfacing technology that can record and stimulate the brain in many different areas with fine spatial and temporal resolution). However, the neuroethical issues and potential tactics for addressing such issues overlap significantly with other spaces (that may encompass this technology or may be tangential to this technology). For example, recent attention to human-centered artificial intelligence has considered potential future scenarios and ethical considerations the overlap with and expand upon some of the concepts identified here (Shneiderman, 2020a,b).

Similarly, many of the ethical concerns and potential approaches involving human enhancement technology apply to the target neurotechnology of the DARPA N^3^ grant. To illustrate, the Sienna Project’s State-of-the-Art Review of Human Enhancement (Jensen et al., 2018), as well as other studies (e.g., Wagner N.-F. et al., 2018) considers Human Enhancement Technology more generally and considers the potential impacts on many different types of parties affected by the technology. However, even the Sienna Project’s Review concludes by setting forth a recommendation that acknowledges a “need for a greater and refreshed dialogue on impacts [of human enhancement technology]., particularly one that looks at specific applications in specific contexts”. While providing projections and potential strategy pertaining to a higher level class of technology can facilitate prudently advancing many technologies, focusing this assessment on a more specific type of potential technology may support more specific analysis of issues and more pertinent potential strategies to employ.

## Conclusion

Just as smart phones and the Internet transformed the way we conduct our lives compared to 20 years ago, brain interfaces 20 years from now may foster more intimate and direct collaborations between brains and technology, allowing augmentation of sensory, motor, communication, and cognitive capabilities. These capabilities may become most utilized across a population when they can be achieved using non-invasive or minutely invasive brain-interfacing technology, and recent research and funding priorities suggest that these types of technology will substantially advance over the next two decades. While previous research has already identified many neuroethical issues that may arise in the future, here we consider a particular hypothetical scenario where there is a high demand in 20 years for non- or minutely invasive brain-recording devices that have capabilities that include at least one of: enhancing attention, memory or learning; enhancing mood; or supporting inter-person communication. We can nonetheless draw from the insightful past work to identify neuroethics issues that may pertain to this potential context and to further identify particular recommendations to address these issues in advance and in real-time. The issues and potential proactive and responsive measures identified here are certainly incomplete, and manufacturer, seller, or user entities may well independently establish anticipatory measures to address potential risks. However, we propose that enacting appropriate ethical frameworks for standards, government programs, oversight, and liabilities will enable the design of ethically guided neurotechnologies that propel humanity to new heights in the near future.

## Author Contributions

KG initiated and supervised the project and coordinated the collection of expert opinions summarized in the figure. HA, KT, PG, JR, PC, and RR led efforts to characterize current capabilities and ongoing research aims of non-invasive and minutely invasive brain-interfacing technology. JI, PC, DE, KG, and AB led efforts to synthesize neuroethics issues and tactics pertaining to non/minutely invasive brain-interfacing technology and to draft the ethical sections. HA and KG led drafting efforts of the regulations and standards section. PG provided expertise in ongoing research aims of non/minutely invasive brain-interfacing technology. DE and AB provided expertise in market forces and business strategies that may influence how brain-interfacing technology develops and is used. All authors contributed to the article and approved the submitted version.

## Conflict of Interest

DE was employed by Neurosensory, Inc. and Braincheck. KG was employed by Kilpatrick Townsend and Stockton LLP. AB was employed by The Substrate Group. KT was employed by Palo Alto Research Center (PARC), A Xerox Company.

The remaining authors declare that the research was conducted in the absence of any commercial or financial relationships that could be construed as a potential conflict of interest.

## Publisher’s Note

All claims expressed in this article are solely those of the authors and do not necessarily represent those of their affiliated organizations, or those of the publisher, the editors and the reviewers. Any product that may be evaluated in this article, or claim that may be made by its manufacturer, is not guaranteed or endorsed by the publisher.
